# Systems Medicine: The Application of Systems Biology Approaches for Modern Medical Research and Drug Development

**DOI:** 10.1155/2015/698169

**Published:** 2015-08-18

**Authors:** Duncan Ayers, Philip J. Day

**Affiliations:** ^1^Centre for Molecular Medicine and Biobanking, University of Malta, Msida MSD 2080, Malta; ^2^Faculty of Medical & Human Sciences, The University of Manchester, Oxford Road, Manchester M13 9PL, UK

## Abstract

The exponential development of highly advanced scientific and medical research technologies throughout the past 30 years has arrived to the point where the high number of characterized molecular agents related to pathogenesis cannot be readily integrated or processed by conventional analytical approaches. Indeed, the realization that several moieties are signatures of disease has partly led to the increment of complex diseases being characterized. Scientists and clinicians can now investigate and analyse any individual dysregulations occurring within the genomic, transcriptomic, miRnomic, proteomic, and metabolomic levels thanks to currently available advanced technologies. However, there are drawbacks within this scientific brave new age in that only isolated molecular levels are individually investigated for their influence in affecting any particular health condition. Since their conception in 1992, systems biology/medicine focuses mainly on the perturbations of overall pathway kinetics for the consequent onset and/or deterioration of the investigated condition/s. Systems medicine approaches can therefore be employed for shedding light in multiple research scenarios, ultimately leading to the practical result of uncovering novel dynamic interaction networks that are critical for influencing the course of medical conditions. Consequently, systems medicine also serves to identify clinically important molecular targets for diagnostic and therapeutic measures against such a condition.

## 1. Introduction

The exponential development of highly advanced scientific and medical research analytical technologies throughout the past 30 years has arrived to the point where most (if not all) key molecular determinants deemed to affect human conditions and diseases can be scrutinized with great detail.

Scientists and clinicians can now begin to attempt investigation of any individual dysregulations occurring within the genomic, transcriptomic, miRnomic, proteomic, and metabolomic levels thanks to advancing wet-lab technologies such as mass spectrometry, quantitative polymerase chain reaction (qPCR) and next generation sequencing, and detailed bioinformatics suites. All these technologies are capable of extracting information from complex datasets to enable disease models to be developed for wet-lab testing. The interplay between the wet and dry lab with specific clinical expertise not only is a main current component of translational medicine, but also is enabled by systems medicine.

However, there are drawbacks within this scientific brave new age, in that in most scientific studies it is only specific molecular levels which are individually investigated for their influence in affecting any particular health condition. Ideally, any form of medical research with the scope of rooting out dysregulated molecular pathway interactions should focus on investigating the holistic aspects of the complex and multifactorial medical condition/s. This involves careful and methodical examination of all simultaneous molecular interactions occurring levels (e.g., genomic, transcriptomic, etc.). Such “bigger-picture” research perspectives lead to a higher level of understanding for complex and multifactorial disease conditions and ultimately “fast-track” the identification and clinical diagnosis of specific molecular pathway dysregulations with pathogenesis value, together with the combined identification of novel drug targets for the development of effective translational therapeutics for the medical condition.

Consequently, the urgent need to counteract such research shortcomings has been acquiesced through the emergence, in the last decade, of the novel research field of systems biology [1, 2].

## 2. Main Principles of Systems Biology Approaches to Research

**Figure 1:**
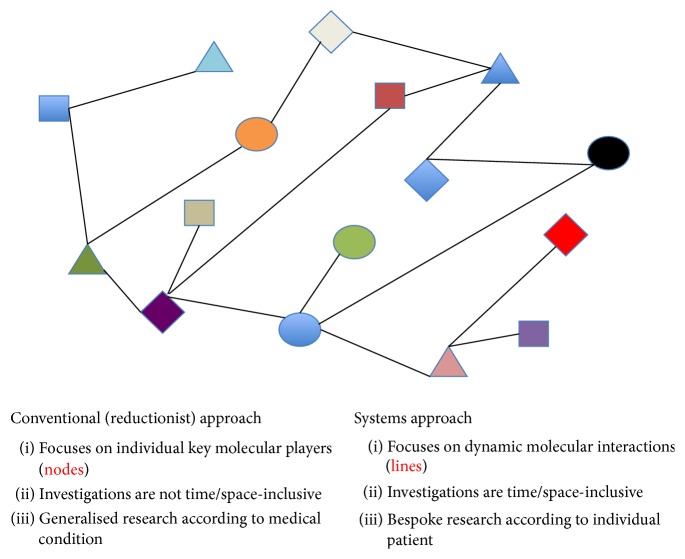
Overview of the main concepts for conventional (reductionist) and systems approaches to modern medical research.

In essence, the field of systems biology revolves around the principle that the phenotype of any individual living organism is a reflection of the simultaneous multitude of molecular interactions from various levels occurring at any one time, combined in a holistic manner to produce such a phenotype (see Figure 1) [3]. Consequently, against the standard concept of reductionist approach where dysregulations in isolated molecular components are studied, data from dysregulations of multiple key molecular players from varying cellular levels of activity are pooled and studied in their entirety, for the purpose of identifying distinct changes in the pattern of intermolecular relationships,* vis a vis* the organism's investigated phenotypes [3, 4]. The methods applied as the principal research tools vary, depending on the nature of the molecular level being investigated and also on the volumes of data generated; therefore, nowadays most self-sufficient systems biology research groups are composed of research scientists with a discernable knowledge of experimental investigation for most molecular level research and/or are unique experts in their own specific research field. Consequently, systems biology is very much an interdisciplinary field of research, requiring the technology platforms and research expertise of individuals from a spectrum of scientific research niche [3, 4]. However, the measurement of all molecular parts of an organ or even biomedical pathway is far from routinely achievable, and great efforts to improve sensitivity of analysis and to make the output data possess a quantitative significance are starting to improve through implementation of field standards [5, 6]. Given current constraints, Boolean approaches are assisting with production of 1st generation systems biology models [7]. A main difference between systems biology and systems medicine is that the former assumes the data to be correct and useable as often wet-lab data generation expertise is not the main goal but is assumed to be correct and useable. Systems medicine (sometimes referred to as systems healthcare) promises to lead with clinical and molecular know-how to produce exquisite datasets that are employed to generate pathway models and treatment and will hopefully directly contribute to stratified medicine en-route to personalized healthcare [4, 8–11].

In addition to performing function as an interdisciplinary research field, systems biology research methods rely heavily on the bioinformatics/computational and mathematical modeling components for achieving answers to the specific research questions [12–18]. Such informatics technology utilization can be twofold in system biology, namely, the implementation of a hypothesis driven “top-down” approach or experimental data driven “bottom-up” approaches [11, 19].

The bottom-up, data driven approach initiates from the collection of large volume datasets derived through a spectrum of omics-based experimental procedures, followed by thorough mathematical modeling analyses to combine the relationships between key molecular players from the varying omics data results obtained [19–21]. One of the primary methodologies employed by the bottom-up systems biology conceptual approach is network modeling [22–25]. A typical biological network model is composed of multiple nodes interacting with each other through edges, whereby nodes are classified as individual key molecular players from any omics level (such as genes, noncoding RNA family members, and proteins) and the edges represent experimentally validated molecular interactions [19]. Both the nature and detail of the nodes and edges within any particular biological network may vary. In addition, highly active nodes interacting in a close-knit network are defined as hubs [26]. Hubs can be further subdivided into two categories, namely, “party” hubs and “date” hubs [26]. Party hubs represent nodes which commonly interact with multiple other molecular partners in a simultaneous manner, whereas date hubs are much more dynamic since they interact with other molecular partners across multiple timeframes and within varying locations [26].

Conversely to the bottom-up experimental methodologies, the hypothesis driven top-down approach relies heavily on mathematical modeling for conducting studies on small-scale molecular interactions for a specific biological condition or phenotype [19, 27]. The dynamical modeling employed for such purpose involves the translation of molecular pathway interactions present in the studied organism into defined mathematical formats, such as ordinary differential equations (ODEs) and partial differential equations (PDEs) that can be analysed and probed within a “dry lab” environment [28–30]. Such a method can be utilized since most intermolecular activities occur with specific kinetics that can be mimicked (e.g., Michaelis-Menten kinetics) by appropriate mathematical derivations [31]. However, dynamical modeling can only be effective if specific assumptions are imposed regarding the biomolecular interactions taking place, such as the selection of defined reaction rate kinetics occurring within the studied biomolecular interactions [31, 32].

In summary, there are four main phases to develop accurately functioning dynamical modeling, namely, model design to identify the pillar intermolecular activities, model construction of such molecular interactions into representative differential equations, model calibration to identify and modulate nonspecific kinetics of individual biomolecular components of the model for the purpose of fine tuning the mathematical model to the experimental format, and model validation by inferring distinct predictions that can be verified in a “wet-lab” experimental scenario [11, 31].

Interestingly, there can also be a third approach to systems biology research models that implement both the top-down and bottom-up methodologies, namely, the middle-out (rational) approach [33, 34].

## 3. Application of Systems Biology for Human Disease: The Advent of Systems Medicine

**Figure 2:**
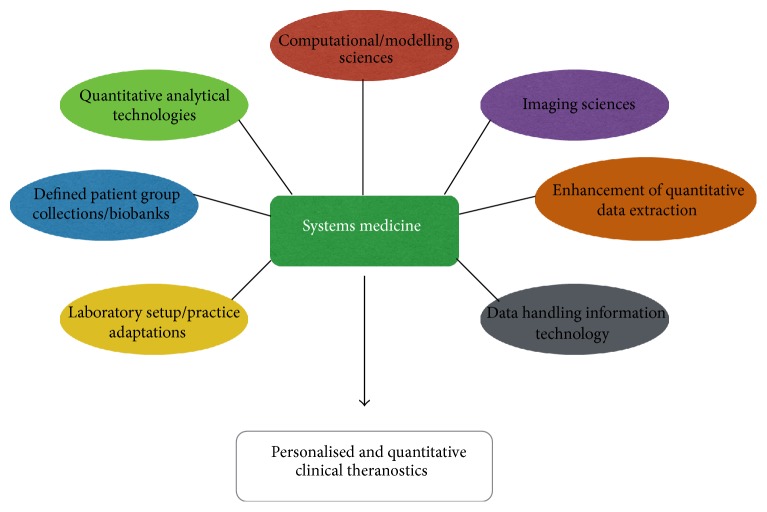
Overview of the required facets for implementation of systems medicine approaches to modern medical research.

The traditional reductionist approach to medical research has been discussed and can be restricted to the investigation of the biological effects of individual or minute quantities of key molecular players for complex, multifactorial human conditions, including cancer. The application of systems biology within the remit of present day medical research can be defined as systems medicine, its concept dating back to 1992 [9]. Such a wider perspective opens new doors of perception and insight into the holistic nature of such disease conditions, focusing mainly on the perturbations of overall pathway kinetics for the consequent onset and/or deterioration of the investigated condition/s. Systems medicine requires the employment of several vital facets in order to attain its clinical theranostic goals whenever such an approach is implemented [35] (see Figure 2).

The essential facets of systems medicine should ideally be established in order to provide proper support for the effective and rapid implementation of any novel research methodologies aimed at reaching the intended outcome for systems medicine-based projects. Undoubtedly, the laboratories involved in conducting systems medicine projects should have the* necessary infrastructure* and research protocol adaptations required for the intense interdisciplinary networking and consequent* data handling and flow of information* that are vital components for enabling successful systems medicine approaches.

Another important component for systems medicine involves the employment of computation of* computational and modeling sciences*. Such expertise is a prerequisite for the effective handling of “big” datasets and also for the interpretation of wet laboratory data in terms of the development of complex interrelationships between varying key molecular players.

Neuroblastoma is the first human condition that has been investigated from a systems level perspective in recent years [19]. Logan et al. constructed a regulatory network model for the main oncogene in neuroblastoma, MYCN, and consequently evaluated the perturbation of this model through the introduction of retinoid drugs (fenretinide, 13-cis-retinoic acid), therefore allowing enhanced insight into the responses of NB tumours to retinoid therapy through the identification of novel molecular interaction hypotheses that can be put to the test in a laboratory setting [19].

The study conducted by Sarmady et al. is apt in demonstrating the versatility of systems-based computational analysis on previously existing experimental data from specific molecular interactions, for the purpose of identifying key molecular players affecting the pathogenesis and severity of the disease condition, in this case Human Immunodeficiency Virus (HIV) [36]. The study applied a motif discovery algorithm on specific groups of HIV viral protein sequences, together with the sequences of immediate binding protein partners found on the host organism [36]. This algorithm ultimately selected only those statistically enriched motifs with conserved viral sequences binding to targeted host proteins [36]. Such an interactome and sequence-based prediction methods allowed for the elucidation of the HIV Nef protein as the main minding site to a multitude of host proteins such as MAPK1, VAV1, LCK, HCK, HLA-A, CD4, FYN, and GNB2L1 [36].

Another example for the use of modeling sciences in systems medicine would be the study conducted by Verma et al., which constructed a systems-based protein regulatory network for the effects of microRNAs (miRNAs) influencing BCR.ABL oncoprotein expression and phosphorylation levels within chronic myeloid leukaemia imatinib-resistant cell line models [37]. This protein regulatory network was deemed to be reliable to identify the varying effects of two specific classes of drugs (tyrosine kinase inhibitors and BCR.ABL-specific miRNAs) on cell lines with differing expression profiles and chemoresistance properties [37]. In addition, for the purpose of this study, quantitative PCR-based high-throughput miRNA expression profiles were established, exemplifying the use of a systems-based approach to develop a protein regulatory network from large scale experimental datasets [37]. This study can also be utilized to illustrate the importance of* quantitative analytical technologies* (in this case, high-throughput RT-qPCR) for driving novel data collection within systems medicine research.

An alternative research scenario in which systems medicine approaches are highly valuable is in the field of biomarker discovery [8]. The recent study carried out by Zhang et al. analysed* in silico* the expression data obtained from high-throughput miRNA and mRNA expression profiling analyses for both primary and metastatic prostate cancer [38]. The results of this analysis highlighted the distinction between two separate miRNA-mRNA correlation and regulatory modular networks for primary and metastatic prostate cancer [38]. This study is a classic example to demonstrate the utility of systems level research for the identification of highly interactive biomarkers delineating differing classes and severity for an individual disease condition that can ultimately serve to monitor (or predict) specific treatment responses in the individual patient.

Another crucial requirement for the successful implementation of systems medicine is the availability of significantly large, though also* highly defined*,* patient groups*. Such patient groups can be organized particularly well if patient samples are provided from curated biobanks.

The study conducted by Albrecht et al. investigated the pathogenesis of hyperuricaemia through the analysis of high-throughput metabolomic profiling data for the regulation of serum urate, which directly induces gout condition in humans and is also associated with cardiovascular disease and diabetes type II [39]. This study employed Gaussian Graphical Modeling with a hypothesis-free approach for the analysis of 355 metabolites from a total of 1764 patients, with the intention to construct a metabolite network affecting serum urate production [39]. The results of this study elucidated a novel serum urate regulatory pathway involving 38 key metabolite components, with a high proportion of such components bearing a gender-specific trait [39].

The study carried out by Mani et al. provides further evidence for the valuable role sustained by adopting a systems level approach for the prediction of oncogenes in cancer conditions [40]. This particular study focused on the analysis of the B-Cell interactome and microarray-based datasets to predict novel oncogenes and molecular perturbation targets, through the identification of dysregulated molecular interactions, in three specific non-Hodgkin's lymphomas [40].

The advances in* imaging sciences* and* quantitative data extraction methodologies* have also been of immense value in attaining successful outcomes through systems medicine approaches. Examples of such technologies include the advent of high content imaging, laser assisted microdissection, and single cell sequencing technology to name but a few [41–43].

Other medical conditions scrutinized through systems level research methodologies include the proliferative fibromatosis condition known as Dupuytren's Disease [44–46] and breast cancer [47] and also within the remit of immunology research [48].

In essence, this change in research perspective by scrutinizing overall molecular network interactions, rather than individual molecules, allows for more effective and clinically applicable research outcomes.

## 4. Systems Medicine Implications in Novel Drug Research and Development

The ever expanding value of systems medicine influences are also currently implemented in order to expedite various aspects of the drug discovery and development protocols within the realm of the pharmaceutical industry.

One of the main research challenges in which systems medicine perspectives can make a major difference is in the prediction of drug adverse effects during the early phases of the drug development process [10]. Pharmacogenetics research for the purpose of drug development has, in the past, focused almost entirely on the effect of variations in individual genes for causing a specific adverse effect [10]. However, such adverse effects are most possibly due to multifactorial influences and as such a systems-based investigation can be far more effective in rooting out and/or predicting harmful adverse effects prior to any novel lead molecule progressing any further within the drug development pipeline [10]. The recent study conducted by Zhao et al. investigated the possibility of identifying a suitable secondary drug to be administered in tandem with the antidiabetic drug rosiglitazone [49]. Rosiglitazone has an associated high risk of myocardial infarction adverse effect; consequently the investigators sought to identify a second drug with the capacity to reduce this risk in patients currently undergoing rosiglitazone [49]. The investigators utilized cell biological network analytical methods on data derived from the Food and Drug Administration's Adverse Event Reporting System (FAERS) together with confirmation of any hypothesis with the use of animal models [49]. This systems-based approach led to the conclusion that exenatide is a suitable drug to administer for minimizing the rosiglitazone cardiac adverse effect risks, through its ability to regulate blood clotting processes [49]. This study demonstrated that apart from playing an important part in predicting drug adverse effects, systems medicine methodologies can also be employed for the prediction of ideal drug combination therapies to be adopted for specific disease conditions.

Another area of drug development in which systems approaches are of significance is in the prediction of drug/target interactions. The study carried out by Babcock et al. investigated drug-induced gene expression profiles for predicting novel inhibitor molecules against the human ether-a-go-go related (hERG) potassium channel, which plays an important part in the regulation of tumour cell proliferation and apoptosis [50, 51]. The study utilized the Connectivity Map (CMap) to select candidate hERG inhibitors with similar gene signature expression profile induction, together with analytical methodologies from databases of experimental datasets for annotated hERG inhibitor activities [50].

Other systems-led methodologies can also be implemented for the purpose of drug/target interaction prediction studies, particularly for large and heterogeneous molecular interaction networks. Such methods include probabilistic matrix factorization and Network-based Random Walk with Restart on the Heterogeneous network (NRWRH) [52, 53]. Similarly, the importance of network pharmacology methods for the identification of novel drug/target networks for individual and/or multiple disease conditions is becoming ever more important due to the recent trend in the application of polypharmacology avenues for maximizing drug development efforts through enhanced treatment successes [54]. This approach is of crucial value particularly for complex and multifactorial clinical conditions such as cancer pathways, bearing a wide spectrum of druggable targets [54].

Systems medicine approaches also play a central role in the emerging drug development area of drug repositioning, whereby drugs deemed to be dated or ineffective for one particular medical condition may however prove to be highly effective for a different condition altogether [55]. The explorative study by Jin et al. focused on the analysis of transcriptomic expression profiles occurring before and after drug administration, as a means of examining and minimizing drug off target effects for major cancer-signaling pathways [56]. This study adopted an integration of one established systems-based analytical approach, namely, Bayesian factor regression model (BFRM), together with the novel cancer-signaling bridges (CSB) network component, with the resultant systems approach termed as CSB-BFRM [56]. The CSB-BFRM was successful in predicting clinical response outcomes for the vast majority of the Food and Drug Administration-approved drugs, with proof-of-concept studies in three separate cancer models (breast cancer, prostate cancer, and leukaemia) confirming the accuracy of the novel systems medicine-based analytical approach [56].

Furthermore, other bioinformatic tools are being developed for aiding researchers to effectively conduct drug repositioning studies. One such tool is the Drugmap Central (http://r2d2drug.org/DMC.aspx), which allows the user to download any multilevel data for established drugs and molecules within one individual framework, in order to enhance swift access to such information [57].

Finally, systems medicine approaches can also have a major impact on the possibilities of identifying novel disease networks, whereby the main investigation focuses on interactions of pathogenesis-influencing molecules that are commonly active and/or dysregulated in multiple disease conditions. A typical example to identify the application of systems medicine methodologies for this research scenario is miRNA research. Since miRNAs regulate transcripts, with one individual miRNA possibly downregulating the expression levels of up to hundreds of downstream target genes, it is no wonder that such miRNAs can be implicated in multiple clinical conditions, possibly in a simultaneous manner [58]. The development of bioinformatics web-tools such as the miRNA BodyMap allows for a clearer visual on the degree of molecular interactions that are directly influenced by miRNA members of the mirnome, therefore easing the task for miRNA researchers to establish the hubs and nodes for their network modeling approaches pertaining to their specific conditions under investigation [58].

## 5. Conclusion and Perspectives

Systems medicine is definitely impacting the way academics, researchers, and clinicians look at medical research experimental approaches. The possibility to simultaneously scrutinize multilevel data from both actual experimental and computational* in silico* sources provides greater insight into the intricate and intertwined, complex molecular interactions. The* interactome* would otherwise remain hidden, as the associations of regulatory processes are not intuitively obvious. This leads to the revealing of novel dynamic interactions that are critical for influencing the course of medical conditions and consequently also serve as clinically important key molecules for future diagnostic and therapeutic agent development.

However, there are still major challenges posing hurdles as emerging technologies such as next generation sequencing and MS provide vast quantities of data and the computational methodologies available at present are only just recently managing to cope with sifting through such high volumes of data to root out meaningful inferences for the posed research queries. In addition, there is no one specific tool that can be utilized to help in the integration of multi-omics datasets, with the results that there is a high degree of subjectivity for the selection of the ideal systems-led approaches.

The future of systems medicine is also shifting its focus onto molecular hubs with a high and versatile influence on the effects of polypharmacology therapies, such as the varying classes of drug transporters within the cellular environment [59, 60]. In addition, systems biology and systems medicine tools should be further implemented and harnessed to achieve the highly ambitious goal of developing the “virtual human” [61], essentially encompassing all the intricate molecular networks and dynamic interactions on multiple omics-levels in order to render the tasks of drug development, through multiple system perturbations with lead molecules, less taxing due to the heavy computational elements provided by such a “virtual human” study model.

In summary, the remaining conceptual shackles restraining the potential of systems-led research, such as the modernizing of the currently acknowledged dogma with Lipinski's Rule of Five for the selection of novel drugs for development [62], together with the traditional “symptom” first for novel drug development (rather than shifting to a “multitarget” first scenario) [59], need to be broken entirely. Additional challenges include the requirement for effective systems medicine models to integrate multiple data masses for accurate identification of novel drug targets, therapies, and also enhanced stratification of patient risk groups. Such effectiveness can only be achieved through proper handling of quantitative data, obtained using vetted and standardized methodologies, with effective data transfer capabilities between multiple software packages and data handling platforms in a smooth manner (as the case is for RDML in handling of RT-qPCR data) [63]. In addition, such data handling should be shared more efficiently across the pharmaceutical industry in order to allow for more rapid theranostic developments.

Ultimately, with the adoption of such novel research perspectives, systems medicine will prove to become one of the mainstays in the way future research will be carried out, not only for extracting further mechanistic knowledge on disease processes but also through a faster and more effective drug development pipeline with the integration of systems-based analysis.
